# [2,7-Dieth­oxy-8-(4-fluoro­benzo­yl)naphthalen-1-yl](4-fluoro­phen­yl)methanone

**DOI:** 10.1107/S1600536813008295

**Published:** 2013-04-05

**Authors:** Saki Mouri, Daichi Hijikata, Katsuhiro Isozaki, Noriyuki Yonezawa, Akiko Okamoto

**Affiliations:** a Department of Organic and Polymer Materials Chemistry, Tokyo University of Agriculture & Technology (TUAT), Koganei, Tokyo 184-8588, Japan; b International Research Center for Elements Science, Institute for Chemical Research, Kyoto University, Gokasho, Uji, Kyoto 611-0011, Japan

## Abstract

In the mol­ecule of the title compound, C_28_H_22_F_2_O_4_, the benzoyl groups are aligned almost anti­parallel and the fluorobenzene rings form a dihedral angle of 14.12 (7)°. The dihedral angles between the 2,7-dieth­oxy­naphthalene ring system and the benzene rings are 70.00 (4) and 67.28 (4)°. In the crystal, mol­ecules are linked by C—H⋯O and C—H⋯F hydrogen bonds, forming layers parallel to the *ab* plane. The layers are further connected by π–π inter­actions [centroid–centroid distances of 3.6115 (10) Å] into a three-dimensional structure.

## Related literature

For electrophilic aroylation of naphthalene derivatives, see: Okamoto & Yonezawa (2009 ▶); Okamoto *et al.* (2011 ▶). For the structures of closely related compounds, see: Nakaema *et al.* (2008 ▶); Watanabe *et al.* (2010 ▶), Isogai *et al.* (2013 ▶).
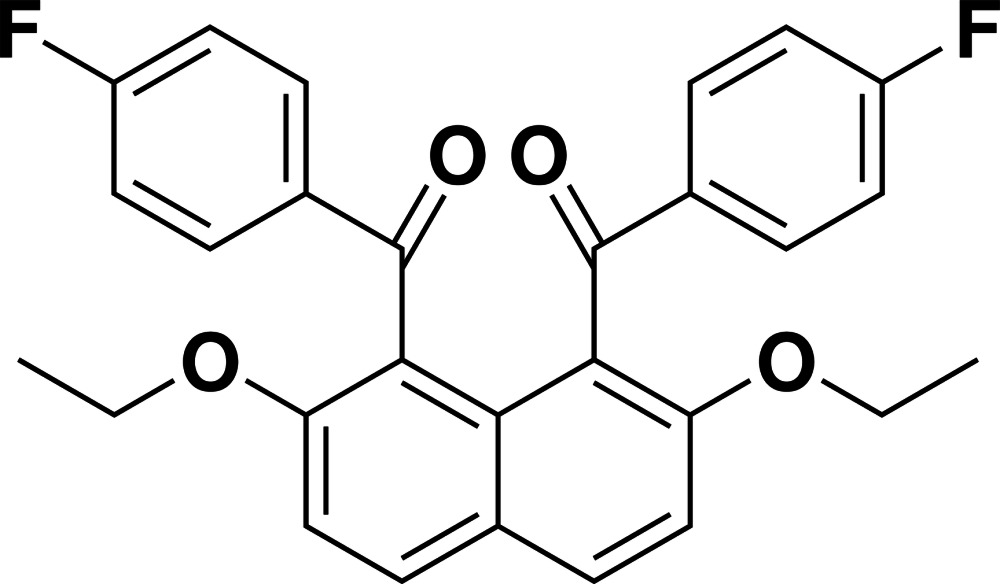


## Experimental

### 

#### Crystal data

C_28_H_22_F_2_O_4_
*M* *_r_* = 460.46
Monoclinic, 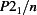

*a* = 7.8592 (18) Å
*b* = 21.243 (5) Å
*c* = 13.941 (3) Å
β = 105.141 (3)°
*V* = 2246.6 (9) Å^3^
*Z* = 4
Mo *K*α radiation
μ = 0.10 mm^−1^
*T* = 173 K
0.20 × 0.20 × 0.20 mm

#### Data collection

Rigaku Saturn70 diffractometer
Absorption correction: numerical (*NUMABS*; Higashi, 1999 ▶) *T*_min_ = 0.980, *T*_max_ = 0.980
14368 measured reflections
3836 independent reflections
3439 reflections with *I* > 2σ(*I*)
*R*_int_ = 0.031

#### Refinement

*R*[*F* ^2^ > 2σ(*F* ^2^)] = 0.036
*wR*(*F* ^2^) = 0.108
*S* = 1.00
3836 reflections
309 parameters
H-atom parameters constrained
Δρ_max_ = 0.20 e Å^−3^
Δρ_min_ = −0.18 e Å^−3^

### 

Data collection: *CrystalClear* (Rigaku/MSC, 2006 ▶); cell refinement: *CrystalClear*; data reduction: *CrystalClear*; program(s) used to solve structure: *Il Milione* (Burla *et al.*, 2007 ▶); program(s) used to refine structure: *SHELXL97* (Sheldrick, 2008 ▶); molecular graphics: *ORTEPIII* (Burnett & Johnson, 1996 ▶); software used to prepare material for publication: *SHELXL97*.

## Supplementary Material

Crystal structure: contains datablock(s) I, global. DOI: 10.1107/S1600536813008295/rz5052sup1.cif

Structure factors: contains datablock(s) I. DOI: 10.1107/S1600536813008295/rz5052Isup2.hkl

Supplementary material file. DOI: 10.1107/S1600536813008295/rz5052Isup3.cml

Additional supplementary materials:  crystallographic information; 3D view; checkCIF report

## Figures and Tables

**Table 1:** Hydrogen-bond geometry (Å, °)

*D*—H⋯*A*	*D*—H	H⋯*A*	*D*⋯*A*	*D*—H⋯*A*
C16—H16⋯O1^i^	0.95	2.30	3.1939 (17)	156
C23—H23⋯O2^ii^	0.95	2.37	3.3214 (18)	176
C6—H6⋯F1^iii^	0.95	2.44	3.1937 (17)	136

Symmetry codes: (i) 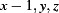; (ii) 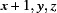; (iii) 
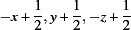
.

**Fig. 1.**
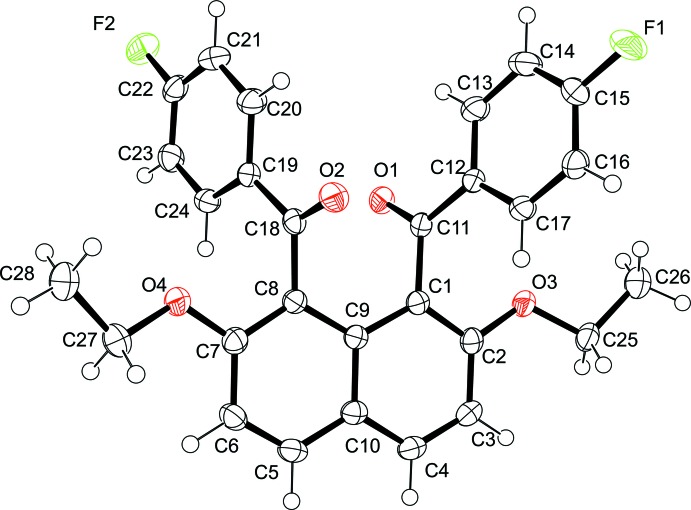
The molecular structure of title molecule, with the atom numbering. The displacement ellipsoids are drawn at the 50% probability level.

**Fig. 2.**
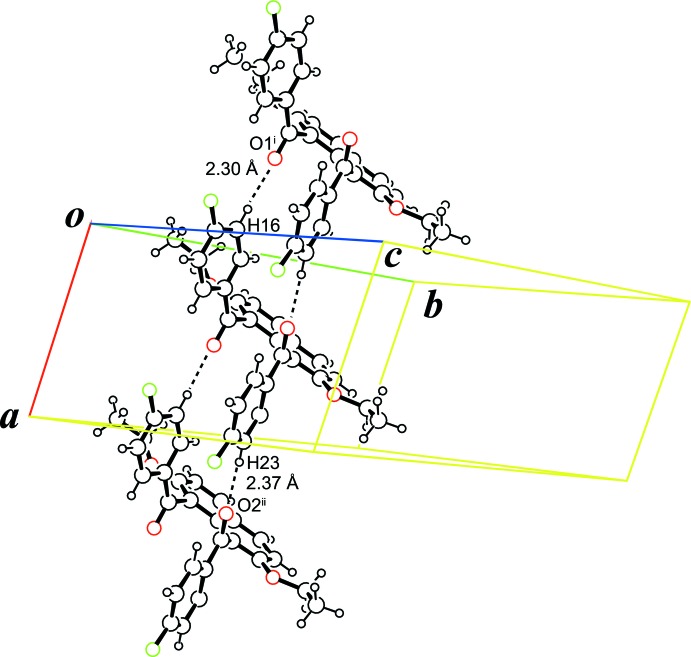
A partial view of the crystal packing of the title compound, showing the intermolecular C—H···O hydrogen bonds (see Table 1 for details; symmetry codes: (i) -1 + x, y, z; (ii) 1 + x, y, z).

**Fig. 3.**
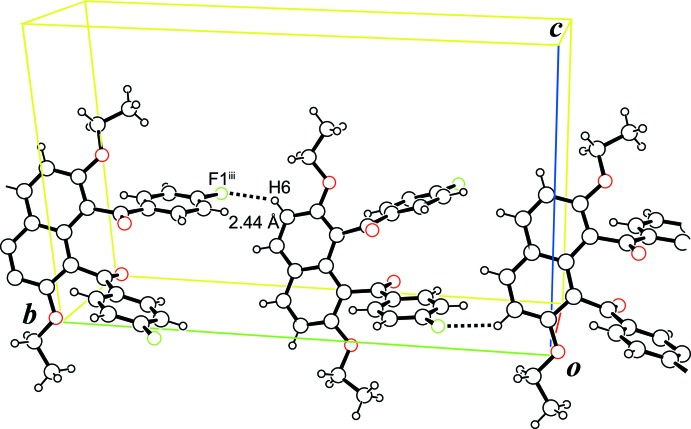
A partial view of the crystal packing of the title compound, showing the intermolecular C—H···F hydrogen bonds (see Table 1 for details; symmetry codes: (iii) 1/2 - x, 1/2 + y, 1/2 - z).

## supplementary crystallographic information

### Comment

In the course of our studies on the selective electrophilic aromatic aroylation of 2,7-dimethoxynaphthalene, *peri*-aroylnaphthalene compounds have proved to be formed regioselectively with the aid of a suitable acidic mediator (Okamoto & Yonezawa, 2009; Okamoto *et al.*, 2011). The two aroyl groups at the 1,8-positions of the naphthalene ring in the resulting compounds are attached in perpendicular fashion to the naphthalene ring and oriented in opposite direction. Recently, we have reported the X-ray crystal structures of 1,8-dibenzoyl-2,7-dimethoxynaphthalene (Nakaema *et al.*, 2008), 1,8-bis(4-fluorobenzoyl)-2,7-dimethoxynaphthalene [(2,7-dimethoxynaphthalene-1,8-diyl)-bis(4-fluorobenzoyl)dimethanone; Watanabe *et al.*, 2010], and 1,8-dibenzoyl-2,7-diethoxynaphthalene [(8-benzoyl-2,7-diethoxynaphthalen-1-yl)-(phenyl)methanone (Isogai *et al.*, 2013). In the crystal of 1,8-dibenzoyl-2,7-dimethoxynaphthalene (Nakaema *et al.*, 2008), the molecule lies across a crystallographic twofold axis so that the asymmetric unit contains one-half of the molecule. The dihedral angle between the benzene ring and the naphthalene ring is 83.59 (5)°. In the case of 1,8-dibenzoyl-2,7-diethoxynaphthalene (Isogai *et al.*, 2013), the dihedral angles are 68.42 (5)° and 71.69 (5)°. On the other hand, two aroyl groups in 1,8-bis(4-fluorobenzoyl)-2,7-dimethoxynaphthalene (Watanabe *et al.*, 2010) are attached to the naphthalene ring with appreciably different dihedral angles of 66.88 (7)° and 88.09 (6)°.

In the crystal packing of 1,8-dibenzoyl-2,7-dimethoxynaphthalene (Nakaema *et al.*, 2008) and analogous compounds (Watanabe *et al.*, 2010, Isogai *et al.*, 2013), the molecules are linked by C—H···O hydrogen bonds between the benzene rings and the ketonic carbonyl groups forming a three-dimensional network. The distances are shorter in the order of 1,8-dibenzoyl-2,7-dimethoxynaphthalene (2.60 Å), 1,8-bis(4-fluorobenzoyl) analogue (2.55 Å), and the 2,7-diethoxy analogue (2.37 Å).

As a part of our continuous studies on the molecular structures of this kind of homologous molecules, the X-ray crystal structure of the title compound, 1,8-bis(4-fluorobenzoyl)-2,7-diethoxynaphthalene is discussed in this article.

In the molecule (Fig. 1), two aroyl groups are non-coplanarly attached to the naphthalene ring and are oriented in opposite direction. The dihedral angles between the planes of the benzene rings [C12—C17 and C19—C24] and the naphthalene ring system (C1—C10) are 70.00 (4)° and 67.28 (4)°, respectively. Besides, the dihedral angle between the benzene rings is 14.12 (7)°.

The molecular packing of the title compound is mainly stabilized by dual C—H···O hydrogen bonds between the benzene rings and the ketonic carbonyl groups along the *a* axis (C16—H16···O1^i^=2.30 Å and C23—H23···O2^ii^=2.37 Å; Table 1 and Fig. 2) and by C—H···F hydrogen bonds between the naphthalene rings and the fluorine atoms on the benzene rings (C6—H6···F1^iii^=2.44 Å; Table 1 and Fig. 3), resulting in the formation of molecular layers parallel to the *ab* plane. In addition, the layers interact through π–π interactions with centroid–centroid distances of 3.6115 (10) Å to form a three-dimensional structure.

The above mentioned results suggest that the title compound has similarities with 1,8-dibenzoyl-2,7-diethoxynaphthalene (Isogai *et al.*, 2013) in the dihedral angles between the benzene rings and the naphthalene ring and the distance of the C—H···O hydrogen bond between the benzene ring and the ketonic carbonyl group.

### Experimental

To a 50 ml flask, 2,7-diethoxynaphthalene (2.0 mmol, 489 mg), 4-fluorobenzoic acid (5.6 mmol, 785 mg), and phosphorus pentoxide–methanesulfonic acid (8.8 ml) were placed. The reaction mixture was stirred at 60°C for 1.5 h. After the reaction, the mixture was poured into ice-cold water and extracted with 25 ml of CHCl_3_ for three times. The combined extracts were washed with water, and 2 *M* aqueous NaOH followed by washing with brine. The organic layers thus obtained were dried over anhydrous MgSO_4_. The solvent was removed under reduced pressure to give cake. The crude product was purified by reprecipitation (CHCl_3_/methanol; isolated yield 91%). Furthermore, the isolated product was crystallized from CHCl_3_ to give single-crystal suitable for X-ray analysis, m.p. 482.7–483.3 K.

### Refinement

All H atoms were located in a difference Fourier map and were subsequently refined as riding atoms, with C—H = 0.95 (aromatic), 0.98 (methyl) and 0.99 (methylene) Å, and with *U*_iso_(H) = 1.2*U*_eq_(C). The positions of methyl H atoms were rotationally optimized.

### Crystal data

**Table:**

C_28_H_22_F_2_O_4_	*F*(000) = 960
*M**_r_* = 460.46	*D*_x_ = 1.361 Mg m^−^^3^
Monoclinic, *P*2_1_/*n*	Mo *K*α radiation, λ = 0.71070 Å
Hall symbol: -P 2yn	Cell parameters from 7582 reflections
*a* = 7.8592 (18) Å	θ = 1.8–31.3°
*b* = 21.243 (5) Å	µ = 0.10 mm^−^^1^
*c* = 13.941 (3) Å	*T* = 173 K
β = 105.141 (3)°	Block, colorless
*V* = 2246.6 (9) Å^3^	0.20 × 0.20 × 0.20 mm
*Z* = 4	

### Data collection

**Table:**

Rigaku Saturn70 diffractometer	3836 independent reflections
Radiation source: fine-focus sealed tube	3439 reflections with *I* > 2σ(*I*)
Graphite monochromator	*R*_int_ = 0.031
Detector resolution: 7.314 pixels mm^-1^	θ_max_ = 25.0°, θ_min_ = 2.9°
ω scans	*h* = −9→9
Absorption correction: numerical (*NUMABS*; Higashi, 1999)	*k* = −25→24
*T*_min_ = 0.980, *T*_max_ = 0.980	*l* = −14→16
14368 measured reflections	

### Refinement

**Table:**

Refinement on *F*^2^	Primary atom site location: structure-invariant direct methods
Least-squares matrix: full	Secondary atom site location: difference Fourier map
*R*[*F*^2^ > 2σ(*F*^2^)] = 0.036	Hydrogen site location: inferred from neighbouring sites
*wR*(*F*^2^) = 0.108	H-atom parameters constrained
*S* = 1.00	*w* = 1/[σ^2^(*F*_o_^2^) + (0.0756*P*)^2^ + 0.3064*P*] where *P* = (*F*_o_^2^ + 2*F*_c_^2^)/3
3836 reflections	(Δ/σ)_max_ < 0.001
309 parameters	Δρ_max_ = 0.20 e Å^−^^3^
0 restraints	Δρ_min_ = −0.18 e Å^−^^3^

### Special details

**Table:**

Geometry. All e.s.d.'s (except the e.s.d. in the dihedral angle between two l.s. planes) are estimated using the full covariance matrix. The cell e.s.d.'s are taken into account individually in the estimation of e.s.d.'s in distances, angles and torsion angles; correlations between e.s.d.'s in cell parameters are only used when they are defined by crystal symmetry. An approximate (isotropic) treatment of cell e.s.d.'s is used for estimating e.s.d.'s involving l.s. planes.
Refinement. Refinement of *F*^2^ against ALL reflections. The weighted *R*-factor *wR* and goodness of fit *S* are based on *F*^2^, conventional *R*-factors *R* are based on *F*, with *F* set to zero for negative *F*^2^. The threshold expression of *F*^2^ > σ(*F*^2^) is used only for calculating *R*-factors(gt) *etc*. and is not relevant to the choice of reflections for refinement. *R*-factors based on *F*^2^ are statistically about twice as large as those based on *F*, and *R*- factors based on ALL data will be even larger.

### Fractional atomic coordinates and isotropic or equivalent isotropic displacement parameters (Å^2^)

**Table:**

	*x*	*y*	*z*	*U*_iso_*/*U*_eq_	
F1	−0.20117 (11)	0.21137 (4)	0.11577 (9)	0.0549 (3)	
F2	1.12357 (11)	0.23089 (4)	0.40563 (7)	0.0453 (2)	
O1	0.52489 (11)	0.33949 (4)	0.12884 (7)	0.0287 (2)	
O2	0.39925 (11)	0.37322 (4)	0.32983 (7)	0.0300 (2)	
O3	0.19889 (11)	0.41165 (4)	−0.05336 (6)	0.0281 (2)	
O4	0.71334 (12)	0.48080 (4)	0.44235 (7)	0.0332 (2)	
C1	0.36340 (14)	0.43375 (6)	0.10775 (9)	0.0207 (3)	
C2	0.27492 (15)	0.45634 (6)	0.01510 (9)	0.0235 (3)	
C3	0.27392 (16)	0.52098 (6)	−0.00825 (9)	0.0261 (3)	
H3	0.2124	0.5357	−0.0723	0.031*	
C4	0.36247 (16)	0.56209 (6)	0.06242 (10)	0.0269 (3)	
H4	0.3626	0.6056	0.0467	0.032*	
C5	0.54467 (17)	0.58524 (6)	0.23023 (10)	0.0279 (3)	
H5	0.5434	0.6286	0.2131	0.033*	
C6	0.63394 (17)	0.56678 (6)	0.32334 (10)	0.0287 (3)	
H6	0.6949	0.5968	0.3704	0.034*	
C7	0.63480 (16)	0.50247 (6)	0.34901 (9)	0.0257 (3)	
C8	0.55115 (15)	0.45756 (5)	0.28119 (9)	0.0216 (3)	
C9	0.45706 (14)	0.47612 (5)	0.18297 (9)	0.0208 (3)	
C10	0.45389 (15)	0.54162 (6)	0.15839 (9)	0.0240 (3)	
C11	0.37995 (15)	0.36319 (5)	0.11976 (8)	0.0210 (3)	
C12	0.22361 (15)	0.32380 (5)	0.11927 (9)	0.0220 (3)	
C13	0.24445 (17)	0.25880 (6)	0.12850 (11)	0.0324 (3)	
H13	0.3575	0.2406	0.1358	0.039*	
C14	0.10147 (19)	0.22059 (7)	0.12717 (13)	0.0412 (4)	
H14	0.1145	0.1762	0.1331	0.049*	
C15	−0.06047 (17)	0.24858 (7)	0.11706 (11)	0.0346 (3)	
C16	−0.08619 (16)	0.31241 (6)	0.10869 (10)	0.0305 (3)	
H16	−0.1995	0.3303	0.1021	0.037*	
C17	0.05794 (16)	0.34984 (6)	0.11013 (9)	0.0260 (3)	
H17	0.0437	0.3942	0.1048	0.031*	
C18	0.54065 (15)	0.39165 (6)	0.32057 (8)	0.0223 (3)	
C19	0.70005 (15)	0.35089 (6)	0.34630 (8)	0.0224 (3)	
C20	0.68344 (17)	0.28956 (6)	0.37819 (10)	0.0307 (3)	
H20	0.5731	0.2755	0.3859	0.037*	
C21	0.82595 (19)	0.24894 (6)	0.39870 (11)	0.0354 (3)	
H21	0.8148	0.2070	0.4199	0.042*	
C22	0.98441 (17)	0.27100 (6)	0.38757 (10)	0.0306 (3)	
C23	1.00746 (17)	0.33129 (6)	0.35714 (10)	0.0312 (3)	
H23	1.1189	0.3452	0.3508	0.037*	
C24	0.86278 (16)	0.37107 (6)	0.33609 (9)	0.0274 (3)	
H24	0.8749	0.4128	0.3143	0.033*	
C25	0.06420 (17)	0.43112 (7)	−0.13949 (10)	0.0302 (3)	
H25A	0.1171	0.4542	−0.1863	0.036*	
H25B	−0.0216	0.4591	−0.1199	0.036*	
C26	−0.0255 (2)	0.37232 (7)	−0.18738 (12)	0.0454 (4)	
H26A	−0.0673	0.3479	−0.1384	0.055*	
H26B	0.0582	0.3470	−0.2121	0.055*	
H26C	−0.1259	0.3837	−0.2429	0.055*	
C27	0.76537 (19)	0.52558 (7)	0.52180 (10)	0.0344 (3)	
H27A	0.6655	0.5536	0.5234	0.041*	
H27B	0.8641	0.5518	0.5127	0.041*	
C28	0.82134 (19)	0.48859 (8)	0.61620 (10)	0.0383 (3)	
H28A	0.8508	0.5175	0.6729	0.046*	
H28B	0.9250	0.4632	0.6154	0.046*	
H28C	0.7249	0.4609	0.6221	0.046*	

### Atomic displacement parameters (Å^2^)

**Table:**

	*U*^11^	*U*^22^	*U*^33^	*U*^12^	*U*^13^	*U*^23^
F1	0.0318 (5)	0.0366 (5)	0.0949 (8)	−0.0117 (4)	0.0141 (5)	0.0159 (5)
F2	0.0370 (5)	0.0385 (5)	0.0562 (6)	0.0174 (4)	0.0046 (4)	0.0041 (4)
O1	0.0185 (4)	0.0284 (5)	0.0384 (5)	0.0045 (3)	0.0059 (4)	−0.0015 (4)
O2	0.0231 (5)	0.0318 (5)	0.0367 (5)	−0.0034 (4)	0.0105 (4)	0.0029 (4)
O3	0.0288 (5)	0.0281 (5)	0.0231 (5)	0.0021 (4)	−0.0008 (4)	0.0001 (3)
O4	0.0414 (5)	0.0268 (5)	0.0251 (5)	0.0018 (4)	−0.0025 (4)	−0.0040 (4)
C1	0.0162 (5)	0.0229 (6)	0.0242 (6)	0.0004 (4)	0.0073 (5)	0.0013 (5)
C2	0.0178 (6)	0.0274 (6)	0.0256 (6)	0.0010 (5)	0.0062 (5)	−0.0011 (5)
C3	0.0242 (6)	0.0279 (6)	0.0262 (6)	0.0053 (5)	0.0067 (5)	0.0060 (5)
C4	0.0260 (6)	0.0229 (6)	0.0331 (7)	0.0032 (5)	0.0102 (5)	0.0063 (5)
C5	0.0291 (6)	0.0192 (6)	0.0365 (7)	0.0005 (5)	0.0104 (5)	−0.0005 (5)
C6	0.0283 (6)	0.0227 (6)	0.0341 (7)	−0.0019 (5)	0.0062 (5)	−0.0064 (5)
C7	0.0231 (6)	0.0259 (6)	0.0272 (7)	0.0022 (5)	0.0048 (5)	−0.0014 (5)
C8	0.0181 (5)	0.0210 (6)	0.0260 (6)	0.0017 (5)	0.0062 (5)	−0.0006 (5)
C9	0.0162 (5)	0.0217 (6)	0.0258 (6)	0.0011 (4)	0.0078 (5)	−0.0002 (5)
C10	0.0211 (6)	0.0222 (6)	0.0303 (7)	0.0016 (5)	0.0094 (5)	0.0016 (5)
C11	0.0190 (6)	0.0232 (6)	0.0198 (6)	0.0016 (5)	0.0035 (4)	−0.0014 (5)
C12	0.0216 (6)	0.0218 (6)	0.0207 (6)	0.0009 (5)	0.0024 (4)	0.0004 (4)
C13	0.0253 (6)	0.0258 (7)	0.0447 (8)	0.0046 (5)	0.0066 (6)	0.0056 (6)
C14	0.0355 (8)	0.0223 (7)	0.0637 (10)	−0.0003 (6)	0.0094 (7)	0.0088 (6)
C15	0.0265 (7)	0.0309 (7)	0.0440 (8)	−0.0084 (6)	0.0052 (6)	0.0077 (6)
C16	0.0207 (6)	0.0326 (7)	0.0369 (7)	0.0014 (5)	0.0054 (5)	0.0052 (6)
C17	0.0229 (6)	0.0222 (6)	0.0324 (7)	0.0018 (5)	0.0065 (5)	0.0021 (5)
C18	0.0229 (6)	0.0244 (6)	0.0190 (6)	−0.0019 (5)	0.0044 (5)	−0.0019 (5)
C19	0.0235 (6)	0.0226 (6)	0.0198 (6)	−0.0005 (5)	0.0034 (5)	−0.0001 (5)
C20	0.0293 (7)	0.0288 (7)	0.0347 (7)	−0.0012 (5)	0.0092 (5)	0.0063 (5)
C21	0.0410 (8)	0.0244 (6)	0.0394 (8)	0.0032 (6)	0.0082 (6)	0.0084 (6)
C22	0.0302 (7)	0.0292 (7)	0.0284 (7)	0.0100 (5)	0.0006 (5)	0.0004 (5)
C23	0.0221 (6)	0.0328 (7)	0.0374 (7)	−0.0005 (5)	0.0057 (5)	−0.0011 (6)
C24	0.0255 (6)	0.0225 (6)	0.0333 (7)	−0.0015 (5)	0.0059 (5)	0.0020 (5)
C25	0.0260 (6)	0.0348 (7)	0.0259 (7)	0.0028 (5)	−0.0003 (5)	0.0029 (5)
C26	0.0436 (8)	0.0419 (9)	0.0395 (8)	−0.0017 (7)	−0.0094 (7)	−0.0002 (7)
C27	0.0387 (7)	0.0339 (7)	0.0285 (7)	−0.0037 (6)	0.0049 (6)	−0.0085 (6)
C28	0.0374 (8)	0.0470 (9)	0.0280 (7)	−0.0011 (6)	0.0041 (6)	−0.0037 (6)

### Geometric parameters (Å, º)

**Table:**

F1—C15	1.3555 (15)	C14—C15	1.378 (2)
F2—C22	1.3571 (15)	C14—H14	0.9500
O1—C11	1.2213 (14)	C15—C16	1.371 (2)
O2—C18	1.2165 (15)	C16—C17	1.3799 (18)
O3—C2	1.3680 (15)	C16—H16	0.9500
O3—C25	1.4385 (15)	C17—H17	0.9500
O4—C7	1.3662 (16)	C18—C19	1.4876 (16)
O4—C27	1.4360 (16)	C19—C24	1.3909 (17)
C1—C2	1.3831 (17)	C19—C20	1.3936 (18)
C1—C9	1.4313 (17)	C20—C21	1.3833 (19)
C1—C11	1.5100 (16)	C20—H20	0.9500
C2—C3	1.4107 (17)	C21—C22	1.377 (2)
C3—C4	1.3636 (19)	C21—H21	0.9500
C3—H3	0.9500	C22—C23	1.3760 (19)
C4—C10	1.4118 (18)	C23—C24	1.3852 (18)
C4—H4	0.9500	C23—H23	0.9500
C5—C6	1.3619 (19)	C24—H24	0.9500
C5—C10	1.4132 (18)	C25—C26	1.502 (2)
C5—H5	0.9500	C25—H25A	0.9900
C6—C7	1.4118 (18)	C25—H25B	0.9900
C6—H6	0.9500	C26—H26A	0.9800
C7—C8	1.3826 (17)	C26—H26B	0.9800
C8—C9	1.4311 (17)	C26—H26C	0.9800
C8—C18	1.5140 (16)	C27—C28	1.497 (2)
C9—C10	1.4316 (17)	C27—H27A	0.9900
C11—C12	1.4851 (16)	C27—H27B	0.9900
C12—C17	1.3896 (17)	C28—H28A	0.9800
C12—C13	1.3925 (18)	C28—H28B	0.9800
C13—C14	1.3823 (19)	C28—H28C	0.9800
C13—H13	0.9500		
			
C2—O3—C25	118.36 (10)	C17—C16—H16	121.1
C7—O4—C27	118.57 (10)	C16—C17—C12	121.19 (12)
C2—C1—C9	120.19 (11)	C16—C17—H17	119.4
C2—C1—C11	117.11 (10)	C12—C17—H17	119.4
C9—C1—C11	122.02 (10)	O2—C18—C19	121.48 (11)
O3—C2—C1	115.60 (11)	O2—C18—C8	118.29 (11)
O3—C2—C3	122.64 (11)	C19—C18—C8	120.23 (10)
C1—C2—C3	121.63 (11)	C24—C19—C20	119.08 (11)
C4—C3—C2	119.05 (11)	C24—C19—C18	122.33 (11)
C4—C3—H3	120.5	C20—C19—C18	118.55 (11)
C2—C3—H3	120.5	C21—C20—C19	120.72 (12)
C3—C4—C10	121.66 (12)	C21—C20—H20	119.6
C3—C4—H4	119.2	C19—C20—H20	119.6
C10—C4—H4	119.2	C22—C21—C20	118.15 (12)
C6—C5—C10	121.71 (12)	C22—C21—H21	120.9
C6—C5—H5	119.1	C20—C21—H21	120.9
C10—C5—H5	119.1	F2—C22—C23	118.49 (12)
C5—C6—C7	119.17 (12)	F2—C22—C21	118.31 (12)
C5—C6—H6	120.4	C23—C22—C21	123.19 (12)
C7—C6—H6	120.4	C22—C23—C24	117.79 (12)
O4—C7—C8	115.77 (11)	C22—C23—H23	121.1
O4—C7—C6	122.58 (11)	C24—C23—H23	121.1
C8—C7—C6	121.62 (12)	C23—C24—C19	121.07 (12)
C7—C8—C9	119.92 (11)	C23—C24—H24	119.5
C7—C8—C18	116.83 (11)	C19—C24—H24	119.5
C9—C8—C18	122.57 (10)	O3—C25—C26	106.83 (11)
C8—C9—C1	124.47 (11)	O3—C25—H25A	110.4
C8—C9—C10	117.98 (11)	C26—C25—H25A	110.4
C1—C9—C10	117.54 (11)	O3—C25—H25B	110.4
C4—C10—C5	120.50 (11)	C26—C25—H25B	110.4
C4—C10—C9	119.92 (11)	H25A—C25—H25B	108.6
C5—C10—C9	119.57 (11)	C25—C26—H26A	109.5
O1—C11—C12	121.09 (11)	C25—C26—H26B	109.5
O1—C11—C1	118.14 (10)	H26A—C26—H26B	109.5
C12—C11—C1	120.77 (9)	C25—C26—H26C	109.5
C17—C12—C13	119.21 (11)	H26A—C26—H26C	109.5
C17—C12—C11	122.03 (11)	H26B—C26—H26C	109.5
C13—C12—C11	118.76 (11)	O4—C27—C28	106.82 (12)
C14—C13—C12	120.36 (12)	O4—C27—H27A	110.4
C14—C13—H13	119.8	C28—C27—H27A	110.4
C12—C13—H13	119.8	O4—C27—H27B	110.4
C15—C14—C13	118.29 (13)	C28—C27—H27B	110.4
C15—C14—H14	120.9	H27A—C27—H27B	108.6
C13—C14—H14	120.9	C27—C28—H28A	109.5
F1—C15—C16	118.28 (12)	C27—C28—H28B	109.5
F1—C15—C14	118.57 (12)	H28A—C28—H28B	109.5
C16—C15—C14	123.15 (12)	C27—C28—H28C	109.5
C15—C16—C17	117.80 (12)	H28A—C28—H28C	109.5
C15—C16—H16	121.1	H28B—C28—H28C	109.5
			
C25—O3—C2—C1	162.02 (10)	C2—C1—C11—C12	−70.49 (14)
C25—O3—C2—C3	−21.99 (16)	C9—C1—C11—C12	118.99 (12)
C9—C1—C2—O3	176.08 (9)	O1—C11—C12—C17	179.27 (11)
C11—C1—C2—O3	5.37 (15)	C1—C11—C12—C17	−1.48 (17)
C9—C1—C2—C3	0.05 (17)	O1—C11—C12—C13	−0.57 (17)
C11—C1—C2—C3	−170.66 (10)	C1—C11—C12—C13	178.68 (11)
O3—C2—C3—C4	−175.76 (11)	C17—C12—C13—C14	0.9 (2)
C1—C2—C3—C4	−0.01 (18)	C11—C12—C13—C14	−179.24 (13)
C2—C3—C4—C10	−0.45 (18)	C12—C13—C14—C15	−0.3 (2)
C10—C5—C6—C7	−0.46 (18)	C13—C14—C15—F1	−179.95 (13)
C27—O4—C7—C8	−165.39 (11)	C13—C14—C15—C16	−0.3 (2)
C27—O4—C7—C6	12.87 (17)	F1—C15—C16—C17	179.97 (12)
C5—C6—C7—O4	−176.44 (11)	C14—C15—C16—C17	0.3 (2)
C5—C6—C7—C8	1.72 (19)	C15—C16—C17—C12	0.31 (19)
O4—C7—C8—C9	176.68 (10)	C13—C12—C17—C16	−0.90 (19)
C6—C7—C8—C9	−1.59 (18)	C11—C12—C17—C16	179.25 (11)
O4—C7—C8—C18	5.96 (16)	C7—C8—C18—O2	106.26 (13)
C6—C7—C8—C18	−172.32 (11)	C9—C8—C18—O2	−64.20 (15)
C7—C8—C9—C1	−178.81 (11)	C7—C8—C18—C19	−74.15 (14)
C18—C8—C9—C1	−8.64 (17)	C9—C8—C18—C19	115.39 (12)
C7—C8—C9—C10	0.23 (16)	O2—C18—C19—C24	179.91 (11)
C18—C8—C9—C10	170.41 (10)	C8—C18—C19—C24	0.33 (17)
C2—C1—C9—C8	179.41 (11)	O2—C18—C19—C20	2.35 (18)
C11—C1—C9—C8	−10.35 (17)	C8—C18—C19—C20	−177.23 (11)
C2—C1—C9—C10	0.36 (16)	C24—C19—C20—C21	−0.56 (19)
C11—C1—C9—C10	170.60 (10)	C18—C19—C20—C21	177.08 (12)
C3—C4—C10—C5	179.97 (11)	C19—C20—C21—C22	0.6 (2)
C3—C4—C10—C9	0.86 (18)	C20—C21—C22—F2	−178.65 (12)
C6—C5—C10—C4	−179.98 (12)	C20—C21—C22—C23	0.0 (2)
C6—C5—C10—C9	−0.87 (18)	F2—C22—C23—C24	178.07 (11)
C8—C9—C10—C4	−179.91 (10)	C21—C22—C23—C24	−0.6 (2)
C1—C9—C10—C4	−0.80 (16)	C22—C23—C24—C19	0.60 (19)
C8—C9—C10—C5	0.97 (16)	C20—C19—C24—C23	−0.05 (19)
C1—C9—C10—C5	−179.92 (10)	C18—C19—C24—C23	−177.59 (11)
C2—C1—C11—O1	108.79 (12)	C2—O3—C25—C26	−166.28 (11)
C9—C1—C11—O1	−61.74 (15)	C7—O4—C27—C28	171.67 (11)

### Hydrogen-bond geometry (Å, º)

**Table:**

*D*—H···*A*	*D*—H	H···*A*	*D*···*A*	*D*—H···*A*
C16—H16···O1^i^	0.95	2.30	3.1939 (17)	156
C23—H23···O2^ii^	0.95	2.37	3.3214 (18)	176
C6—H6···F1^iii^	0.95	2.44	3.1937 (17)	136

Symmetry codes: (i) *x*−1, *y*, *z*; (ii) *x*+1, *y*, *z*; (iii) −*x*+1/2, *y*+1/2, −*z*+1/2.
